# Feedback delays can enhance anticipatory synchronization in human-machine interaction

**DOI:** 10.1371/journal.pone.0221275

**Published:** 2019-08-22

**Authors:** Auriel Washburn, Rachel W. Kallen, Maurice Lamb, Nigel Stepp, Kevin Shockley, Michael J. Richardson

**Affiliations:** 1 Center for Computer Research in Music and Acoustics, Department of Music, Stanford University, Stanford, CA, United States of America; 2 Department of Psychology, Center for Elite Performance, Expertise and Training, and Perception in Action Research Center, Macquarie University, Sydney, NSW, Australia; 3 Center for Cognition, Action and Perception, Department of Psychology, University of Cincinnati, Cincinnati, OH, United States of America; 4 HRL Laboratories, LLC, Malibu, CA, United States of America; Lanzhou University of Technology, CHINA

## Abstract

Research investigating the dynamics of coupled physical systems has demonstrated that small feedback delays can allow a dynamic response system to anticipate chaotic behavior. This counterintuitive phenomenon, termed *anticipatory synchronization*, has been observed in coupled electrical circuits, laser semi-conductors, and artificial neurons. Recent research indicates that the same process might also support the ability of humans to anticipate the occurrence of chaotic behavior in other individuals. Motivated by this latter work, the current study examined whether the process of feedback delay induced anticipatory synchronization could be employed to develop an interactive *artificial agent* capable of anticipating chaotic human movement. Results revealed that incorporating such delays within the movement-control dynamics of an artificial agent not only enhances an artificial agent’s ability to anticipate chaotic human behavior, but to synchronize with such behavior in a manner similar to natural human-human anticipatory synchronization. The implication of these findings for the development of human-machine interaction systems is discussed.

## Introduction

Cyber-based social interaction and coordination has become increasingly ubiquitous in our society due to rapid advances in online social networking, interactive *virtual-reality (VR)* systems, and *human-machine interaction (HMI)*. Effective cyber-based collaboration depends on the ability of both human and artificial agents to behave in a highly flexible and mutually responsive manner, constantly adapting to what each other is doing or will do next. HMI technologies are currently limited in the degree to which they can synchronize with and anticipate human actions during complex time-evolving behaviors [1–4]. As such, the capabilities of interactive artificial agents are not currently comprehensive of the coordinative characteristics essential to daily human activity. Specifically, co-acting individuals frequently synchronize their movements together, often also anticipating or leading one another’s movements during coordinated interaction. Overcoming this limitation is key to the development of safe and successful HMI for perceptual-motor development, training and rehabilitation, social skills training and daily activity assistance, and tele-present human-machine interaction and industrial human-robot construction [5, 6].

There is a common assumption in cognitive science and psychology that feedback delays within the human perceptual-motor system (i.e., the combined input + output + input latencies of the peripheral and central nervous systems), negatively affect human behavior by amplifying perceptual-motor errors [7, 8]. As a result, neural simulation processes [9–10], feed-forward internal models [8, 11], or shared intentional and representational states [12] have been hypothesized to account for how the human nervous system compensates for such delays. However, recent research exploring human movement synchronization has revealed that small (millisecond) perceptual-motor feedback delays might actually facilitate, rather than hinder, the ability of human actors to both synchronize with and anticipate the continuous actions of others, even when the behavior involves chaotic action sequences [13, 14]. This counterintuitive phenomenon is referred to as *anticipatory synchronization* and occurs when a human actor is attempting to coordinate with the continuous movements of an environmental stimulus or another individual, while experiencing small *visual feedback delays* (VFD) between the production and the perceived outcome of their movements [8, 13–14]. These VFDs correspond to the difference in time between when a user’s motor action occurs and their visual perception of that motor action or the motor action’s outcome.

**Fig 1:**
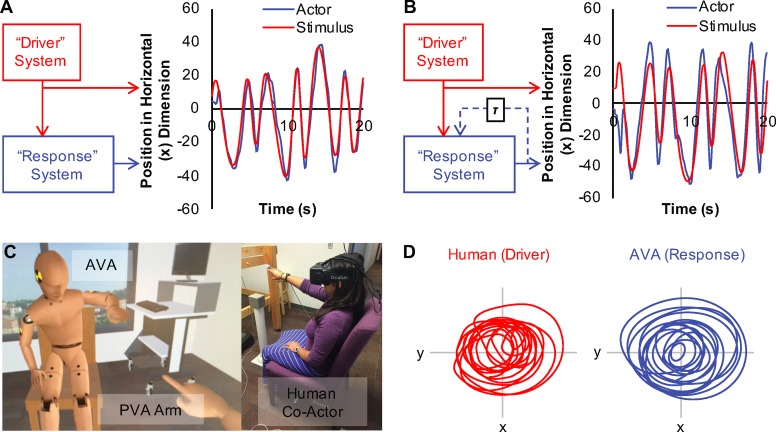
Coupled response-driver systems. Response-driver coupling is depicted schematically (A), along with time series from one dimension of coordinated stimulus (red) and participant (blue) movements (B) (adapted from [10] and previously demonstrated in [9]). Examples of (A) non-anticipation and (B) anticipatory synchronization are provided. The example of non-anticipation comes from a trial in which the participant experienced no feedback delay, and the example of anticipatory synchronization from a trial in which the participant experienced a 400 ms visual-motor feedback delay. Similar results were observed during interpersonal interaction with a weak bi-directional coupling between individuals. (C) The participant’s view within the virtual environment (left) and the general experimental set-up for the current study (right). (D) Typical movement time series for the AVA (left) and human co-actor (right) from AVA testing trials; i.e., human acting as driver and AVA as the response system.

The phenomenon of anticipatory synchronization was actually first discovered in research examining coupled chaotic physical systems, such as coupled laser semiconductors [15, 16], coupled electrical circuits [17], and coupled neurons [18]. This research revealed that when a “response” system (i.e., electrical circuit) is coupled to a chaotically behaving “driver” system (i.e., a second electrical circuit) and small temporal feedback delays are introduced into the response system, the response system begins to anticipate the behavior exhibited by the chaotic driver system (see Fig 1A and 1B). In short, the feedback delay essentially couples a past state of the response system to the current state of the driver system, resulting in the response system’s current state moving temporally contiguous with and then ahead of the driver’s current state. Without the inclusion of these delays the response system’s behavioral state will always exhibit a temporal lag with respect to the chaotic behavior of the diver system.

With regard to human behavior, Stepp and colleagues [8, 13, 19, 20] first investigated whether the process of anticipatory synchronization might also underlie human anticipatory coordination with chaotic environmental behavior. To evaluate this, [13] instructed participant responders to coordinate the movements of a dot-stimulus displayed on a computer screen, that they controlled by moving a hand-held stylus across a touch sensitive tablet, with the continuous movements of a second, computer driven dot stimulus. The movement dynamics of the computer driver stimulus were defined by a chaotic Rössler spring system (see 13–15 for more details). During the coordination task, participants experienced a set of small VFDs between their actual hand/stylus movements and the outcome of their movements represented by the stimulus dot they controlled on the computer screen. The authors used a cross-correlation analysis to establish the temporal relationship associated with the greatest level of synchronization between the movements of the participant responder and the motion of the computer driven stimulus. The results of this analysis revealed that while the participant lagged behind the computer driven stimulus in the absence of any feedback delay (i.e., VFD ≈ 0 ms), VFDs between 100–400 ms resulted in the participant movements leading the motion of a chaotic, computer-driven stimulus (i.e., participants exhibiting anticipatory synchronization).

Washburn and colleagues [14] then extended the research on human anticipatory synchronization by investigating the ability of a responder participant to coordinate with the chaotic movements produced by a second, driver participant under various VFD conditions. From this point on we will use the term ‘driver’ to refer to the corresponding participant or agent who is producing the target movement and the term ‘responder’ to refer to the participant or agent who is attempting to synchronize their movements with those of the driver. In [14], the driver participant practiced generating chaotic arm movements by observing and coordinating with a visual stimulus controlled by the same chaotic Rössler spring system employed by [13]. Notably, biological movement is often inherently chaotic [21]. This movement practice was therefore not meant to teach participants a novel type of movement pattern, but rather to achieve some experimental consistency among participants with regards to the size, shape, and speed of chaotic action sequences performed during the study.

The driver participant then used a hand-held motion-tracking sensor to produce chaotic visual stimulus movements that were displayed to the participant responder via a large television monitor. The responder also controlled a visual stimulus using a hand-held motion-tracking sensor, with small VFDs (between ≈ 0 to 600 ms) introduced between the responder’s real arm movements and the corresponding motion of their visual stimulus. Consistent with anticipatory synchronization, responders maintained a strong level of synchronous coordination for delays up to 400 ms, but more importantly, began to produce movements that anticipated and spatiotemporally lead the chaotic movements of the driver for the 200 ms VFD condition. Note that this anticipatory synchronization occurred even when the responder’s movements were also observable to the driver participant, indicating that the emergence of anticipatory synchronization is not dependent on unidirectionally coupled “driver-responder” systems, but can also occur when the driver and responder are bidirectionally coupled.

It is important to appreciate that the experimentally induced VFDs employed by [13, 14] were superimposed on top of the feedback delays already inherent to the human sensorimotor system. In other words, the experimentally induced VFDs extended the length of existing delays associated with signal transmission between the neural activity that occurs in preparation for movement, the movement itself, and the perceptual outcomes associated with the movement. For example, in [13, 14] the VFDs were an additive result of the natural human VFD, motion-tracking/controller latency, system processing time, and the display refresh rate of the display system employed, with the latter three delays equaling between 25 to 35 ms. These feedback delay manipulations therefore offer an exaggerated view of the naturally occurring anticipatory synchronization processes that result from pre-existing neurocognitive delays. Indeed, the fact that participants are able to naturally synchronize with chaotic movements produced by environmental stimuli or other human actors at all, even with a small lag, is indicative of anticipatory behavior [13, 14, 19].

Of particular interest in the current study was whether the introduction of VFDs within the control architecture of an artificial agent could induce (simulate) a comparative anticipatory synchronization process within an HMI task context. More specifically, the aim of the current study was to investigate whether it is possible to leverage the functional understanding of human anticipatory synchronization to enhance the degree of anticipatory synchronization exhibited by artificial agents engaged in HMI. In order to accomplish this, we developed a time-delayed coupled, “humanoid” *artificial virtual avatar* (AVA) whose “task” was to coordinate its end-effector (hand and corresponding forearm) movements with the chaotic forearm movements of human participants. It is important to keep in mind that not all of the actions or movements produced by human actors during interpersonal or multiagent interaction are chaotic. However, it is not uncommon for continuous human movements to exhibit aperiodic patterns of behavioral variability consistent with chaotic processes (e.g., [21]). Moreover, given the aims of the present study, the unpredictable nature of chaotic movement patterns means that such movements are the optimal movements for determining the occurrence of anticipatory synchronization [13, 14].

In our study seated human participants viewed the AVA seated directly in front of and facing them within a simple virtual environment via an Oculus Rift VR head-mounted-display. The participants were also represented as a virtual avatar (*participant virtual avatar* or PVA) within the VR environment viewed using the VR headset and were instructed to generate chaotic, elliptical movements (see Fig 1D), which were captured in real-time by means of a hand-held motion tracking sensor and depicted in the forearm movements of the PVA (see Fig 1C). Our general expectation was that short VFDs (implemented as time-delayed coupling functions) within the movement control architecture of the artificial virtual agent would significantly improve the ability of the agent to anticipate the chaotic movements of human co-actors, or at a minimum significantly reduce the asynchrony that occurred between the AVA and the participant-*driver*.

## Method

### Experimental design

#### Participants

Seventeen University of Cincinnati undergraduate students (between 18 to 31 years of age) participated in the study. This sample size was consistent with previous work on anticipatory synchronization in human subjects [13, 14].

The study was approved by the University of Cincinnati Institutional Review Board (IRB) (protocol #2012–2827). Participants were recruited using the University of Cincinnati Psychology Research Participation System. All participants provided written informed consent. The individual pictured in Fig 1A of this manuscript has given written informed consent (as outlined in the PLOS consent form) to publish the image.

#### Apparatus

We employed a virtual reality (VR) interface in the current study. This afforded us the opportunity to examine the phenomenon of human-machine anticipatory synchronization within a realistic, yet highly controllable setting. It should be noted that prior to conducting the study described here, a preliminary study examining anticipatory synchronization between an AVA-*driver* and a participant-*responder* was conducted to validate the VR paradigm employed. The results of this preliminary study (see Supplementary Material 1) replicated the results of [14], demonstrating that small (ms) VFDs can induce anticipatory synchronization in (human) participant responders and, moreover, that anticipatory synchronization can emerge when the coupling between driver and responder is both uni- and bi-directional.

For the current study, participants were asked to sit, wearing an Oculus Rift VR headset in order to interact with a simple virtual environment generated using Unity 3D. The environment included the AVA seated directly in front of and facing the participant, as well as a PVA, whose location was mapped to that of the participant’s seated location (see Fig 1C). Participants controlled the right end-effector of the PVA via a Polhemus motion tracking sensor attached to the first two fingers of their right hand. The forearm and upper arm movements of the PVA’s right arm were generated through an inverse kinematics controller (Final IK: an inverse kinematics solution for Unity game developers, developed by Partel Lang).

We used a Polhemus Liberty electro-magnetic motion capture system (~0.1 mm accuracy) (Polhemus Liberty, Polhemus Corporation, Colchester, VT) to track participants’ movements at 120 Hz. The horizontal and vertical coordinates of participant movement were also recorded from the magnetic tracking system through the Unity game engine at a sampling rate of 75 Hz (it was these latter time series that were used for analysis). The Polhemus motion-tracking receiver was positioned approximately 10 cm in front of the fingers of a participant’s right arm outstretched directly in front of their body. The minimum visual feedback delay latency with respect to participants’ real arm movements and the movements of their PVA within the VR environment was used during all participant movement familiarization trials and AVA testing trials. Due to the time necessary to achieve movement tracking (~5.32 ms) and data transfer (~5 to 8 ms) and the head mounted display refresh rate (~13.33 ms), the minimal delay between a participant’s movement and the rendering of that movement within the visual scene took up to 26.67 ms.

#### Participant (driver) movement practice

Before testing whether small VFDs could induce anticipatory synchronization in the AVA, participants completed two movement practice trials. In these trials, participants practiced generating chaotic forearm movements by synchronizing the movements of their virtual avatar arm with the chaotic forearm movements produced by the AVA. As in [14], we conducted this practice exercise to establish experimental consistency among participants’ with regards to the chaotic variability of movement form, size and shape.

During these practice trials, the participants’ task was to synchronize their PVA arm movements with the 2-D movements of the AVA’s left arm for 100 s. The 100 s movements of the AVA were generated ahead of time (i.e., pre-recorded), such that the movements of the participant had no influence on the movements of the AVA (i.e., the coupling was uni-directional; participant-*response* coupled to AVA-*driver*). These pre-recorded movement sequences were generated by the following chaotic spring system,

$${\dot{x}}_{1}=x_{2}+(p_{1}-x_{1})$$

$${\dot{x}}_{2}=-{\left(\omega \pi (\frac{x_{3}}{\alpha }+\beta )\right)}^{2}x_{1}+(p_{2}-x_{2})$$

$${\dot{x}}_{3}=-x_{4}-x_{5}$$

$${\dot{x}}_{4}=x_{3}+\alpha x_{4}$$

$${\dot{x}}_{5}=b+x_{5}(x_{3}-c)$$ (1)

where the *x*_*3*_, *x*_*4*_ and *x*_*5*_ dimensions define a standard Rössler attractor [13]. This attractor gives rise to the chaotic dynamics that define position in the horizontal and vertical dimensions for a simple harmonic oscillator, specified by *x*_1_ and *x*_2_ and respectively. The chaotic spring system produces a generally elliptical trajectory over time. However, the amplitude and frequency of the system behavior are transient and the trajectory diverges rapidly from stable harmonic/periodic movements, resulting in a chaotic trajectory which is not readily predicted by a participant (see Fig 1D).

All participants experienced the same two chaotic AVA movement sequences, with the order of presentation alternated across participants. These sequences were determined from those sequences employed for participant practice in [14] and that [14] found consistently led to participants producing chaotic movement behavior when they were later asked to take on a *driver* role. As in [14], Largest Lyapunov Exponent Analysis (LLE) was employed to index the chaotic nature of the movement sequences, with movement sequences that produced positive LLEs containing chaotic movement dynamics; see [22] and Statistical analysis section for further details). The LLEs for the two movement sequences employed were 0.273 and 0.257, respectively, with the average movement frequency of these two trials equaling .31 Hz (Seq. 1: *M* = .311, *SD* = .075; Seq. 2: *M =* .316, *SD* = .075). Note that this movement frequency was similar to the independent movement frequency of participant-*drivers* in [14], where the overall average frequency was .32 Hz.

#### AVA (response) to participant (driver) testing

For the testing trials, participants were informed that they would be switching roles with the AVA. That is, participants were told that they would be the leader (i.e., driving movement producer) and that the AVA would be attempting to synchronize with their chaotic movements. The participants could always see AVA behavior during this task, resulting in a constant bi-directional coupling between the AVA-*response* and participant-*driver* systems, and were asked to continue creating the same kinds of 2-D movements they had been producing during the movement practice trials. More specifically, they were instructed to move their forearm in a manner that was “generally circular, but unpredictable in terms of the speed and amplitude of movement” (see Fig 1D for example participant-*driver* movement time series).

While a variety of candidate systems exist for defining the baseline behavioral dynamics of an AVA like the one designed in the current study, a harmonic spring system was selected based on the fact that it is highly flexible and has relatively few intrinsic dynamics compared to other candidate response systems. For a response system with inherently chaotic dynamics it would be harder to evaluate whether anticipatory behavior of a chaotic driver system was primarily a product of coordination or simply the response system’s naturally emerging behavior. The harmonic spring system used to define the baseline movement of the AVA’s end-effector (hand and corresponding forearm movements) during the testing trials took the form

$${\dot{x}}_{1}=x_{3}+C_{R-D}(D_{1}-x_{1d})$$

$${\dot{x}}_{2}=x_{4}+C_{R-D}(D_{2}-x_{2d})$$

$${\dot{x}}_{3}=-\omega x_{1}$$

$${\dot{x}}_{4}=-\omega x_{2}$$ (2)

where the horizontal and vertical dimensions of AVA movement are defined by *x*_*1*_ and *x*_*2*_, respectively.

Here, the coupling function *C*_*R-D*_(D_*_−x_*d_), where *R-D* corresponds to *response-to-driver*, is of primary significance, in that *C*_*R-D*_ acts to modulate the strength of AVA coupling to the horizontal, *D*_*1*_, and vertical, *D*_*2*_, dimensions of continuous participant-*driver* movement. The coupling achieved during interaction with a participant was therefore a *response system* (AVA) to *driver system* (participant) coupling, hereafter referred to as *R-D coupling*. With the incorporation of time-delays within the AVA response system this form of coupling is known as *time*-*delay coupling*. This coupling references the horizontal and vertical dimensions of the AVA’s own past behavior, *x*_*1d*_ and *x*_*2d*_, with respect to the participant-*driver’s* current corresponding behavioral states [20, 23–25]. The past behavior being accounted for within the delay-coupling, *x*_*d*_, is always the position of the system at a set length of time, τ, prior to the current time point, *t*,

$$x_{d}=x(t-\tau ).$$ (3)

As a result, the values of *x*_1d_ and *x*_2d_ are the values of *x*_1_ and *x*_2_ at (*t—*τ). The variable τ thus comes to express a self-referential feedback delay within the AVA system, allowing us to test the effects of a range of delay latencies on facilitating synchronization and anticipation by the AVA with respect to chaotic human behavior. The harmonic spring oscillator system defining AVA behavior in the current experiment can therefore be understood to approximate key sensorimotor processes involved in previous demonstrations of human anticipatory synchronization.

In preliminary model simulations (see S1 Appendix) a coupling strength of *C*_*R-D*_ = 1.75 resulted in anticipatory behavior by the system defining AVA-*response* behavior that was most similar to human anticipatory synchronization [13, 14]. During data collection the value for AVA *C*_*R-D*_ was treated as a randomly assigned, between-subjects variable such that participant*-drivers* either interacted with the AVA coupled to them at a lower (*C*_*R-D*_ = 1.5) or higher (*C*_*R-D*_ = 2.0) strength. Eight participants experienced the lower coupling strength and nine participants experienced the higher coupling strength. There were no significant differences between the results observed for the two different coupling strengths and thus the results presented in the current paper have been collapsed across this factor. As a result, the R-D coupling strength in this experiment had an average value of 1.75.

Five different values of self-referential delay latency, τ, were implemented within the AVA-*response* system (26.67, 106.64, 199.95, 306.59, and 399.90 ms). We selected these delays as those achievable within the VR setup that were most similar to the delays that induced anticipatory synchronization during model simulation with the system defining AVA-*response* behavior (see S1 Appendix). Each of the five delays were instituted once per participant, with the order of presentation randomized over the five test trials experienced by each participant.

The position variables of the harmonic spring system defining AVA-*response* behavior, *x*_*1*_ and *x*_*2*_, interact with the term ω in this system to determine spring stiffness, affecting the average movement frequency of the AVA. In the current study the spring stiffness term within the AVA system, ω, was set to a value of 1.87. This was determined by

$$\omega =\frac{2\pi }{T}.$$ (4)

Here *T* is very close to the average period of oscillation for movements produced during the two movement familiarization trials (i.e. 3.36 s, equivalent to a movement frequency of .30 Hz). We expected participants to generate movements of a similar speed during the testing trials. In reality, the average participant-*driver* movement frequency for a given AVA testing trial ranged from .22 to .54 Hz.

#### Procedure

Participants were informed that the study was designed to test the ability of a virtual agent to coordinate with natural human movement. Following task instructions, participants then completed the two participant-*response* to AVA-*driver* practice trials. They then completed the five AVA-*response* to participant-*driver* testing trials, after which they were debriefed and thanked for their participation. The full experiment took approximately 20–30 minutes.

### Data reduction and analysis

Prior to analysis, the first 10 s and last 5 s of each 60 s time series were discarded to remove transients. The resulting 45 s time-sires were also low-passed filtered using a 10Hz, 4^th^ order Butterworth filter prior to analysis to remove noise.

#### Largest Lyapunov Exponent (LLE)

LLE indexes the attractor dynamics of a time series, with a positive LLE indicative of chaotic dynamics [22]. We analyzed the LLEs for participant-*driver* and AVA-*response* movement sequences in the AVA testing trials of the current study. This allowed us to establish that on average the participant-*driver* behavior was chaotic during the AVA testing trials, and to evaluate the dynamics of the resulting AVA coordinative movement. In order to determine the LLE of each participant and AVA time series we conducted an analysis based on the algorithm developed by [26]. For this analysis, the time series for the vertical and horizontal dimensions of actor behavior were initially treated separately. In the current study the same patterns were observed in both dimensions for each of the actor movements examined, and were therefore averaged to establish characteristic LLE values for the AVA and participant movement dynamics exhibited in each trial.

#### Maximum cross-correlation

Maximum cross-correlation provides a measure of the overall synchronization between two time series over a specified range of temporal relationships. In the current study this allowed us to establish (i) the maximum level of synchrony achieved between AVA-*response* and human*-driver* movements for each trial (i.e., the maximum cross-correlation coefficient) as well as (ii) the degree to which the movements of the AVA lagged or led those of the participant-*driver* (i.e., specific temporal relationship between the AVA and participant associated with the maximum cross-correlation coefficient).

The cross-correlation between the AVA and participant movement time series for a given trial was calculated using the equation

$$xcorr(h)=\frac{\sum_{i}\left[(a(i)-\bar{a})(p(i-h)-\bar{p})\right]}{\sqrt{\sum_{i}({a(i)-\bar{a})}^{2}}\sqrt{\sum_{i}{\left(p(i-h)-\bar{p}\right)}^{2}}}.$$ (5)

Here, the *a* and *p* variables correspond to AVA and participant positions, respectively, and *i* and *h* refer to the current and shifted time points, respectively, such that *x*corr(*h*) represents the normalized cross-correlation function of the two time series at a temporal shift equal to *h*. For each trial, the value of the cross-correlation between the two time series was calculated for a range of temporal shifts of the AVA time series with respect to the participant time series, extending 2 s ahead of and 2 s behind perfect synchrony (*h* = [–150, 150]). From the resulting cross-correlation function, the maximum cross-correlation value, [i.e., max *x*corr(h)] and its associated temporal shift value, *h*, were employed to index the degree of synchrony observed between the AVA and participant and at what lag/lead relationship this synchrony occurred, respectively. In short, for the current study, a max *x*corr(h) = 0 indicated the complete absence of synchrony between the AVA and participant at the given temporal lag/lead, whereas a value of 1 indicated perfect synchrony between the AVA and participant at a given lag/lead. With respect to the corresponding value of *h* (i.e., the AVA lag/lead with respect to the participant), negative values indicated lagging behavior by the AVA and positive values indicated AVA leading behavior, or anticipation.

This same cross-correlation analysis was conducted for both the horizontal and vertical dimensions of movement. As the same patterns were observed for both the horizontal and vertical dimensions, these values were then averaged to determine a single maximum cross-correlation and AVA lag/lead measure for each trial.

#### Instantaneous Relative Phase (IRP)

IRP captures the spatiotemporal patterning of coordination between two contemporaneous, oscillatory time series [27, 28], and was used here to determine the (i) mean and (ii) standard deviation (SD) of the relative phase between the AVA-*response* and the participant-*driver* over the course of a trial, as well as (iii) the frequency distribution of relative phase relationships visited. Each of these three measures required us to first compute the continuous phase angle series for the vertical and horizontal dimensions of the recorded participant and AVA time series, and then from the continuous phase time series calculate the corresponding instantaneous relative phase angles that occurred between the AVA and participant for a given movement dimension.

Here we employed the Hilbert transform to first compute separate continuous phase angle series, θ(*t*), for each movement times-series dimension, where

$$\theta (t)=arctan\left(\frac{s(t)}{Hs(t)}\right).$$ (6)

Here, s(t) corresponds to the real part of the analytic signal produced by the Hilbert transform and Hs(t) corresponds to the imaginary part of the signal. Following this, the instantaneous relative phase, ϕ(*t*)_,_ between the AVA and participant movements for a given dimension (i.e., vertical or horizontal) were calculated as

$$\phi (t)=\theta_{1}(t)-\theta_{2}(t)$$ (7)

with θ_1_(*t*) and θ_2_(*t*) representing the continuous phase angles of the AVA and participant time-series, respectively. As with the cross-correlation analysis above, the same patterns of relative phase were observed for the vertical and horizontal movement dimensions. Thus, the resulting relative phase values were averaged across the horizontal and vertical dimensions to establish characteristic relative phase measures for each trial.

A constant IRP value of 0° corresponds to perfect inphase synchrony, with both movements simultaneously in the same phase of their respective oscillatory cycles over time. Note that the stability of the IRP relationship between two movements is inversely related to the magnitude of the SD of IRP (i.e., the lower the SD of IRP, the more stable the pattern of coordination defined by mean IRP). Accordingly, a mean and SD of IRP close to 0° for a given trial in the current study would indicate that the movements of the AVA-*response* system were closely synchronized (inphase) with the movements of the participant-*driver*. Perhaps more importantly, mean IRP angles less than or greater than 0° (when the SD of IRP is close to zero), would indicate AVA lagging and/or leading behavior, respectively.

In addition to examining the mean and SD of IRP, the pattern of spatiotemporal coordination between two movement time-series can also be revealed by examining the distribution of IRP values. Indeed, for the current study the average IRP frequency distribution for a given trial allowed us to observe how often each of a range of IRP relationships was achieved, with peaks in a distribution representing the relative stability of the various IRP relationships visited (higher peaks = greater stability). These frequency distributions were calculated in terms of 72 relative phase regions/bins (-180°-180°, in 5° increments). By including positive and negative IRP values in these distributions it was possible to identify the presence of frequent leading or lagging by the AVA-*response* system.

In order to evaluate whether a significant proportion of a trial was spent at a given relative phase relationship we generated 1,000 random IRP time series with the same length (60 s) and sampling rate (75 Hz) as the experimental trials. This allowed us to construct surrogate, random IRP distributions. In each IRP bin the 950^th^ highest proportion of a surrogate series was identified. The largest of these values across the bins was employed as the statistical threshold value based on its correspondence to a .05 significance level [29, 30].

## Results

### Manipulation check

In order to establish that participant*-drivers* and the AVA-*response system* exhibited chaotic movement dynamics during testing, we determined the LLE for participant and AVA movement time series in each trial. This measure provided an index of each behavioral system’s attractor dynamics [22], with a positive LLE indicative of chaotic dynamics (see Method section for further details). We observed average positive LLE values across the five AVA time-delay conditions for both the participant-*driverse* and the AVA, indicating that they generally produced chaotic movement patterns during the testing trials (see Table 1). We used five one sample t-tests to assess whether the average LLE for all participant-*drivers* in each VFD condition were significantly greater than zero. This allowed us to confirm that average participant-*driver* LLEs were significantly greater than zero in all conditions (all *t*(17) > 1.80, *p* < .05) except for the minimum VFD (*t*(17) = 1.25, *p* = .11). In the minimum VFD condition slightly less than 50% of the individual participant-*driver* LLEs were positive, whereas more than 50% of the individual LLEs were positive in each of the other VFD conditions (26.67 ms: 47.06%, 106.64 ms: 70.6%, 199.95 ms: 64.71%, 306.59 ms: 76.47%, 399.90 ms: 58.82%).

**Table 1:** AVA testing trials: Driver and response LLE values.

	AVA Feedback Delay									
	26.67 ms		106.64 ms		199.95 ms		306.59 ms		399.90 ms	
Actor	*M*	*SD*	*M*	*SD*	*M*	*SD*	*M*	*SD*	*M*	*SD*
Participant-*driver*	.026	.085	.020	.048	.053	.108	.025	.057	.047	.079
AVA-*response*	.093	.125	.098	.094	.080	.079	.167	.135	.158	.115

### AVA (response) to participant (driver) coordination

Due to technical problems, one trial from one participant was not recorded for the AVA-*response* system testing trials. Mean substitution for the associated delay condition (106.64 ms VFD condition) was used to replace this cell for statistical analysis.

#### Maximum cross-correlation

**Fig 2:**
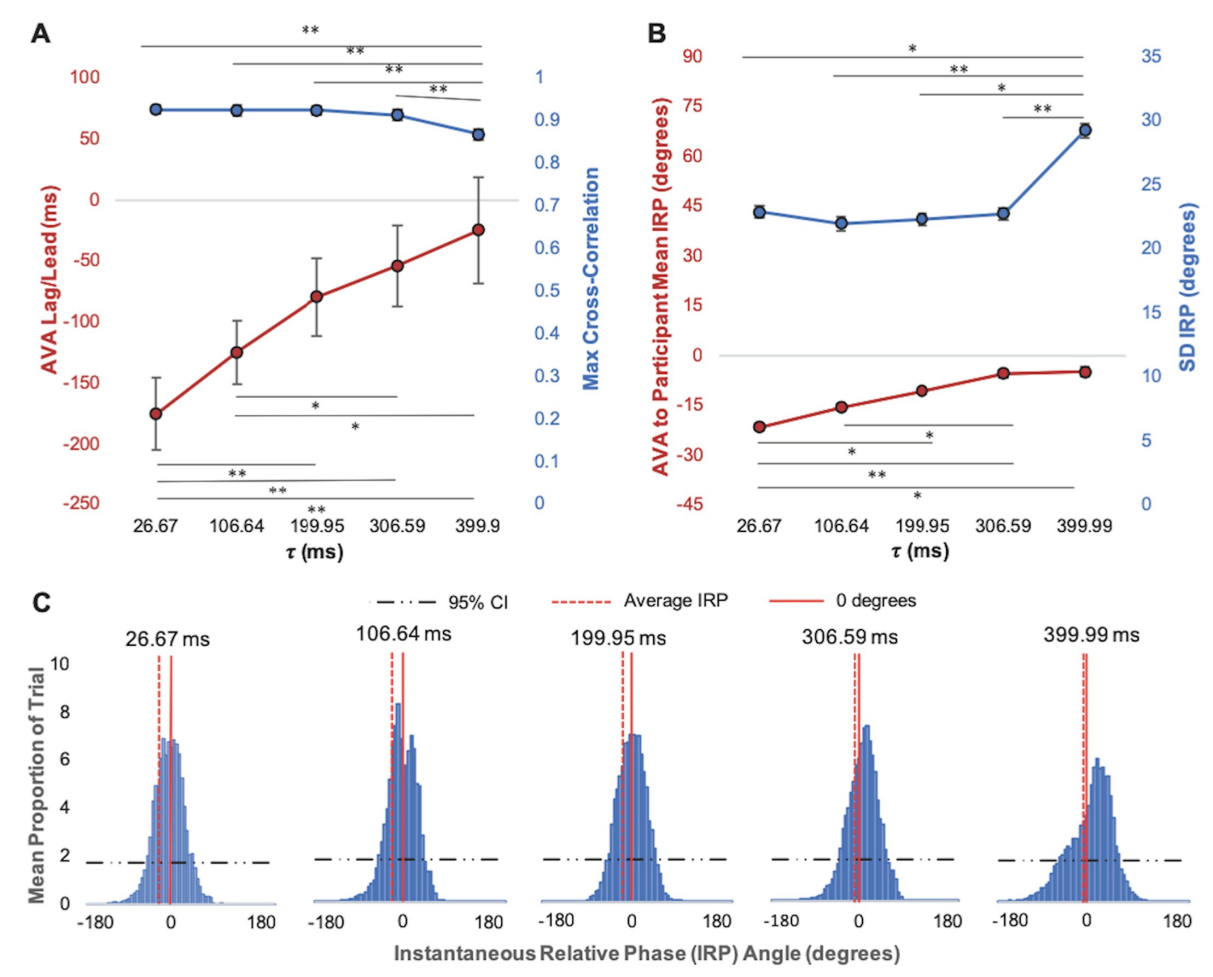
AVA anticipatory synchronization during real-time coordination with a participant-*driver*. (A) Average maximum cross-correlation (blue) and associated AVA lag/lead (red) and (B) Average IRP (red) and standard deviation of IRP (blue) between AVA-*response* system and participant-*driver* movements in each of the feedback delay conditions. Error bars show standard error. *p < .05, **p < .01. (C) Average IRP distributions between AVA and participant-*driver* movements for each feedback delay condition. The mean (average) relative phase angle for each condition, as well the cutoff for the 95% CI corresponding to statistically significant a proportion of time spent at a given IRP relationship are also shown.

Consistent with the previous research on human anticipatory synchronization [13, 14], the analysis of maximum cross-correlation revealed a slight decrease in coordination with increases in feedback delay. A one-way, within-subjects analysis of variance (ANOVA) comparing the maximum cross-correlation between the AVA-*response* and participant-*driver* movements across the five different delay latencies revealed that the effect of feedback delay was indeed significant, *F*(4, 64) = 16.97, *p* < .001, η_p_^2^ = .52 (see Fig 2A). Furthermore, post-hoc comparisons using a Bonferroni correction revealed a significant difference in average maximum cross-correlation between the longest feedback delay (399.90 ms) and each of the four other feedback delay conditions (*p*s < .01).

A one-way, within subjects ANOVA for the effect of delay latency on the AVA’s temporal lag/lead associated with the maximum cross-correlation between AVA and participant-*driver* in each trial also revealed a significant effect of feedback delay, *F* (4, 64) = 16.20, *p* < .001, η_p_^2^ = .50. As can be seen in Fig 2A, the AVA lead became less negative with increases in VFD (i.e., the lag between participant-*driver* and AVA-response behavior decreased)., with the AVA nearing a 0-lag relationship with the participant-*driver* at the 399.90 ms delay latency. Post hoc comparisons with a Bonferroni correction demonstrated significant differences in temporal asynchrony between the 26.67 ms feedback delay and the 199.95, 306.59, and 399.90 ms feedback delay conditions (*p*s < .01), as well as between the 106.64 ms feedback delay condition and each of the 306.59 and 399.90 ms feedback delay conditions (*p*s < .05).

#### IRP

Similar to the temporal AVA lag/lead associated with the maximum cross-correlation analysis, a one-way, within-subjects ANOVA assessing the mean IRP between the AVA-*response* and participant-*driver* systems also showed a significant effect of feedback delay, *F*(4, 64) = 7.98, *p* < .001, η_p_^2^ = .33. As in the maximum cross-correlation analysis, the AVA to participant-*driver* phase lag decreased with increases in feedback delay (see Fig 2B). Post hoc comparisons with a Bonferroni correction showed a significant difference in mean IRP between the shortest feedback delay (26.67 ms) and the 199.95, 306.59, and 399.90 ms delay conditions, as well as between the 106.64 and 306.59 ms delay conditions (*p*s < .05).

A one-way, within-subjects analysis of variance (ANOVA) assessing the standard deviation of IRP values between the AVA and participant also revealed a significant effect of feedback delay, *F*(4, 64) = 8.79, *p* < .001, η_p_^2^ = .36. As shown in Fig 2B, we saw a general increase in relative phase variability with increases in feedback delay. Post hoc comparisons with a Bonferroni correction showed a significant difference in the standard deviation of IRP between the longest feedback delay (399.90 ms) and each of the other delay conditions (26.67, 106.64, 199.95, 306.59 ms) (*p*s < .05).

The average distribution of IRP values visited over the course of a trial were examined separately for all feedback delay latencies (see Fig 2C). At the minimum feedback delay of 26.67 ms the predominant relative phase peak was associated with lagging behavior, but at a feedback delay of 106.64 ms, two distinct peaks revealed intermittent leading and lagging behavior by the AVA-*response* system with respect to the participant-*driver*. Here the peak associated with lagging was still higher than that associated with leading behavior, but the peaks were much more similar in height than at the 26.67 ms feedback delay. In the 199.95 ms feedback delay condition there was a shift to a predominant peak representing AVA leading behavior very close to synchrony. A similar peak persisted for the 306.59 and 399.99 ms feedback delay conditions, continuing to shift toward greater anticipation by the AVA with increases in feedback delay.

The effects of VFD on the IRP distributions were consistent with those identified by the maximum cross-correlation and mean and SD of IRP analyses reported above. Namely, the distributions indicate that the movements of the AVA often lagged behind those of the participant-*driver*. The distribution for the longest feedback delay (399.99 ms) was also associated with decreased stability of the phase relationships achieved, consistent with the statistical analysis of the standard deviation of IRP reported above. It is important to note, however, that the IRP distributions also revealed the presence of true AVA anticipatory behavior for the 106.64 to 399.0 ms feedback delay conditions (i.e., peaks in the IRP distribution above 0°).

## Discussion

The current study was designed to investigate whether the phenomenon of feedback delay induced anticipatory synchronization could be leveraged for adaptive HMI. To achieve this aim, an artificial virtual agent (AVA) was created, whose end-effector (hand) and corresponding arm movements were controlled by a time-delayed, low-dimensional dynamical model coupled to the real-time movements of a human participant producing chaotic (i.e., complex; unpredictable) movement sequences. Of particular interest was whether small self-referential feedback delays introduced into the control architecture of the artificial agent would not only reduce the degree to which the movements of the AVA system lagged behind those of the participant-*driver*, but result in the kind of intermittent phase leading behavior synonymous with human-human anticipatory synchronization [14].

Consistent with these expectations and human anticipatory synchronization more generally [8, 19, 20], an analysis of the maximum cross-correlation and the mean and SD of relative phase observed between the movements of the AVA and participant-*driver* revealed that the introduction of small (ms) self-referential feedback delays within the AVA to participant coupling function did in fact reduce the degree to which the AVA lagged behind the human participant. For feedback delays less than 300 ms, this induction of more synchronous behavior also occurred without any functional effects on coordination stability. In other words, the serviceable stability of the synchronous coordination was largely invariant across the 26.67 to 306.59 ms feedback delay conditions.

These findings were also in line with recent work on modelling the effects of feedback-delayed visual-motor tracking [25]. One prediction from this work that was supported in the current study was that anticipation time increases (i.e., lag time decreases) in a linear fashion with increases in feedback delay up to a certain critical delay after which tracking performance deteriorates. Another prediction generated by [25]’s model was that the maximum temporal anticipation exhibited by the feedback-delayed system cannot exceed half the magnitude of the feedback delay. Both of these predictions were upheld in the real-time test of the AVA in the present work, as well as in the preliminary simulations conducted on this system with pre-recorded human behavior as the driver (see S1 Appendix).

More importantly, however, an examination of the distributions for IRP revealed the presence of anticipatory synchronization with regard to the behavior of the AVA. Specifically, the IRP frequency distributions revealed that the AVA exhibited intermittent leading behavior. This intermittent behavior was similar in form to the patterns of anticipatory synchronization behavior observed in previous human-human research [14] (also see the S1 Appendix). The pattern was most notable at the 100 ms delay condition, although the occurrence with which the AVA exhibited relative phase leading also increased without a consequential effect on coordination stability for the 199.95 and 306.59 ms feedback delays conditions.

In regard to the limitations of the current study, it should be noted that while participant-*driver* behavior was associated with positive average LLE values during the AVA testing trials, some individual trials were associated with negative LLE values. This indicates that participant-*driver* behavior in these trials did not technically exhibit chaotic dynamics, which can be attributed in part to the fact that the participant-*drivers* likely exhibited some adaptation to the AVA during interaction. However, we did establish that the average LLE for each condition was significantly different from zero at all time-delays except for the minimum delay (26.67 ms). We therefore maintain that the AVA behavior observed in the current study effectively demonstrates the ability of artificial agent systems containing self-referential feedback delays to support reduced lagging and the anticipation of chaotic human behavior.

The occurrence and stability of anticipatory behavior by the AVA was also less pronounced compared to other investigations of human-human [14] and participant-*response* to AVA-*driver* (see S1 Appendix) anticipatory synchronization. This may have been affected by participant-*driver* adaptation to the AVA as well. It is important to appreciate, however, that a rather extensive body of previous research has demonstrated that spontaneous interpersonal coordination is often characterized by intermittent, or relative, coordination (e.g., [27]; also see [4, 31, 32]). In fact, intermittent, or relative, coordination is actually characteristic of weakly coupled physical or biological limit-cycle oscillators in general [33]. Thus, the current findings actually suggest that the AVA is capable of achieving a coordinative dynamic that will likely seem natural to human co-actors during bi-directional coupling. Our present results, along with those of [14], indicate that individuals instructed to act as driving systems are able to maintain a low-strength coupling to the behavior of the coordinating response system. However, it is still possible that bi-directional coupling could reduce the independence of participant-*driver* behavior and consequently disrupt AVA-*response* system anticipation. Thus, for situations in which artificial agent anticipation of chaotic human behavior is of greater importance than bi-directional agent-human interaction, a uni-directional R-D coupling between the artificial agent-*response* and human-*driver* systems might be more effective in supporting artificial agent anticipation.

Finding that self-referential feedback delays can support anticipatory synchronization in artificial agents constitutes a substantial contribution to the development of interactive artificial agents and HMI. Indeed, the ability to develop artificial agents capable of producing adaptive, anticipatory behavior during real-time human interaction by means of simple feedback delay mechanisms has potentially transformative implications for achieving more efficient HMI across a wide variety of task contexts. This includes, but is not limited to, perceptual-motor assistance, training, and rehabilitation, social skills training, and industrial assembly.

Having generated an artificial agent capable of anticipatory synchronization using a low-dimensional, coupled dynamical model demonstrates how such bio-inspired task/behavioral dynamics modeling approaches (e.g., [34–39]) can capture universal properties characteristic of many kinds of coupled physical systems, including the human biological systems that underlie the emergence of chaotic neural and perceptual-motor behavior [21, 36, 39–41]. Notably, human biological behavior is often also characterized by regular, periodic dynamics and although time-delay induced anticipatory synchronization may seem less necessary with regard to such movements, such coupling should still have a beneficial effect (all-be-it very modest) . In contrast, the majority of models aimed at depicting more complex dynamics rely on explicit feedforward motor programs, or motor templates. These models may accurately reflect elements of human movement anticipation. However, their effectiveness depends on extensive prior knowledge in order to predict and achieve a specific action. It is possible that coupled dynamical models that do not require prior knowledge, like the one we have presented here, can also account for this behavior. In other words, low-dimensional physical systems may be sufficient to account for anticipatory synchronization, decreasing the need for resource-demanding, high-level processes.

The low-dimensional nature of dynamical systems like the one developed here also allows them to be easily implemented within artificial agent systems. The system controlling our AVA-*response* behavior, and described in Eq 2, can be introduced within any artificial agent system and used to generate continuous 2-D response system behavior synchronized to any 2-D, aperiodic driver system output without any need for training. The AVA-*response* system also produces human-like intermittent anticipatory synchronization, which may be capable of eliciting increases in interpersonal rapport and social awareness that are known to result from synchronous human perceptual-motor interaction (e.g., [42, 43]). Accordingly, a productive avenue for future work is determining whether artificial agents capable of adaptively anticipating complex human behavior can engender the same kinds of positive social outcomes associated with naturally occurring human interaction.

Finally, determining whether feedback delay-induced anticipatory coordination processes can also be transferred to non-periodic (i.e., non-limit-cycle), discrete HMI movement tasks (e.g., object pick-and-place or passing tasks) is also an important avenue for future research. Even within the context of human-human interaction it remains to be seen whether self-referential feedback delays can enhance anticipation behavior during discrete task behavior. Although we are not advocating that all human anticipatory behavior is founded on the feedback delay induced processes of anticipatory synchronization, it is possible that similar processes could also support anticipatory behavior during discrete perceptual-motor behaviors that are deterministic and dynamical and occur over a relatively fast event or time scales (i.e., over seconds or minutes). If true, such findings would further validate the facilitative role of perceptual-motor feedback delays for social coordination and behavioral anticipation more generally. Such findings would have a transformative impact on the scientific understanding of human neural and sensorimotor information processing as well. They would also serve to establish an entirely new theoretical account, based on the lawful dynamics of coupled complex systems, for how human or artificial actors are able to flexibly adapt to the chaotic and context-sensitive behavioral actions of other con-specifics and environmental events.

## Supporting information

S1 Appendix Preliminary paradigm validation and artificial agent simulation. We conducted an initial experiment in order to validate that the virtual reality setup employed for the primary study could be used to investigate the phenomenon of anticipatory synchronization. In preparation for the real-time AVA testing we carried out in the primary study, we also completed a preliminary assessment of the AVA response system through simulation.
(PDF)
