# Supplementary material for: Feedback delays can enhance anticipatory synchronization in human-machine interaction
Source: PLoS One. 2019 Aug 22;14(8):e0221275. doi: 10.1371/journal.pone.0221275 (PMC6705796; doi:10.1371/journal.pone.0221275)
Supplement: S1 Appendix — We conducted an initial experiment in order to validate that the virtual reality setup employed for the primary study could be used to investigate the phenomenon of anticipatory synchronization. In preparation for the real-time AVA testing we carried out in the primary study, we also completed a preliminary assessment of the AVA response system through simulation. (PDF) [file pone.0221275.s001.pdf]

S1 Appendix.

Preliminary paradigm validation and artificial agent simulation

Table of Contents

1. Virtual Reality Paradigm Validation 2

Participants 2

Procedure 2

Table S1.1. Settings for Real-time Generation of AVA-driver Movement. 4

Table S1.2. VR Paradigm Validation: AVA-driver LLE Values 5

Results 5

Fig S1.1. Human-response anticipatory synchronization during VR paradigm testing 6

Conclusions 8

2. Artificial Virtual Avatar (AVA) Response System Simulations 10

Table S1.3. Pre-Recorded Human-driver LLE Values 10

Fig S1.2. AVA-response anticipatory synchronization during simulation with pre-recorded aperiodic human-driver movement. 11

Results 12

Conclusions 13

1. Virtual Reality Paradigm Validation

We conducted an initial experiment in order to validate that the virtual reality (VR) setup we employed for the primary study could be used to investigate the phenomenon of anticipatory synchronization. More specifically, we sought to replicate the findings of previous research investigating human-environment and human-human anticipatory synchronization within a non-VR setting (*13, 14*). We achieved this by assessing whether small *visual feedback delays* (VFDs) could facilitate a human actor's ability to anticipate the movements of a chaotically behaving *artificial virtual agent* (AVA) within a virtual-reality environment (i.e., human-*response* to AVA-*driver* anticipatory synchronization).

Participants

Five undergraduate students and seven graduate students from the University of Cincinnati participated in this experiment along with four other individuals recruited from the greater Cincinnati area, for a total of 16 participants. Participants ranged in age from 19 to 31 years.

The study was approved by the University of Cincinnati Institutional Review Board (IRB) (protocol #2012-2827). Participants were recruited using the University of Cincinnati Psychology Research Participation System. All participants provided written informed consent.

Procedure

The same virtual environment, virtual avatars, and motion tracking system used for the primary study were employed for this paradigm validation experiment (see the Method section of main text for details). Similar to the participant (driver) movement practice trials reported in the main text, response participants in this paradigm validation were instructed to synchronize their hand/arm

movements, or more specifically the movements of their participant virtual avatar (PVA), with those of the AVA's hand/arm movements in a mirroring fashion. The behavior of the AVA system was generated in real time by means of the following chaotic spring system,

$$\begin{aligned}
\dot{x}_1 &= x_2 + C_{D-R}(p_1 - x_1) \\
\dot{x}_2 &= -\left(\omega\pi\left(\frac{x_3}{\alpha} + \beta\right)\right)^2 x_1 + C_{D-R}(p_2 - x_2) \\
\dot{x}_3 &= -x_4 - x_5 \\
\dot{x}_4 &= x_3 + \alpha x_4 \\
\dot{x}_5 &= b + x_5(x_3 - c)
\end{aligned} \qquad \text{(A)}$$

This system is almost identical to the system used to generate the pre-recorded AVA-*driver* movement sequences during the participant movement practice trials in the primary study. In this study, however, this system was employed to generate the AVA arm movements online by controlling the 'x' and 'y', horizontal and vertical, coordinates of the AVAs end-effector (i.e., virtual hand). The AVA's end-effector movements were also coupled in real-time to those of the coordinating human participant by the inclusion of the driver-response (D-R) coupling term, C_{D-R}. This resulted in an influence of each of the 'x' and 'y' coordinates of the participant's end-effector (finger/hand) movements (i.e., p_1 and p_2, respectively) on the 'x' and 'y' coordinates of the AVA end-effector movements (i.e., x_1 and x_2, respectively). Note that for both the PVA and the AVA, we employed the same inverse kinetics engine (i.e., Final IK) used in the primary study to control avatar upper-arm and shoulder movements as a functional end-effector location (see the Method section in main text for more details).

Three different values of the D-R coupling term were tested as distinct within-subjects coupling conditions, providing an opportunity to compare the occurrence and stability of human

anticipatory synchronization in the context of no (0), low (.025) and high (.05) D-R coupling. D-R coupling strength was always set to $C_{D\text{-}R} = 0$ for the first seven seconds in order to allow the chaotic spring system that defined the end-effector movements of the AVA-*driver* to begin to generate chaotic movement behavior at the beginning of each trial. After this period the coupling was adjusted to the value associated with that trial. Nine unique sets of system parameters and initial conditions were selected based on their ability to produce 60 s of bounded chaotic behavior at a D-R value of $C_{D\text{-}R} = 0$ (see Table S1.1). Similar to the chaotic movements examined in (*14*), the AVA-*driver's* average movement frequency for a given trial ranged from .23 to .30 Hz.

Table S1.1. Settings for real-time generation of AVA movement.

	Initial Conditions										Parameter Settings					
Set	x_1	x_2	x_3	x_4	x_5	$\dot{x}_1$	$\dot{x}_2$	$\dot{x}_3$	$\dot{x}_4$	$\dot{x}_5$	a	b	c	α	β	ω
1	14.1	14.23	-7.9	1.12	0.005	15.18	-40.14	-1.32	-7.734	-0.004	0.1	0.1	14	160	0.2	2.3
2	14.1	14.23	-7.9	1.12	0.005	15.18	-40.14	-1.32	-7.734	-0.004	0.1	0.1	14	150	0.23	2
3	14.1	14.23	-7.9	1.12	0.005	15.18	-40.14	-1.32	-7.734	-0.004	0.1	0.1	14	150	0.22	2
4	-18.3	-6.9	-6.9	0	9.102	-17.9	-17.56	-9.23	-0.002	-5.753	0.1	0.1	14	160	0.23	2
5	-18.3	-6.9	-6.9	0	9.102	-17.9	-17.56	-9.23	-0.002	-5.753	0.1	0.1	14	160	0.2	2.3
6	-18.3	-6.9	-6.9	0	9.102	-17.9	-17.56	-9.23	-0.002	-5.753	0.1	0.1	14	160	0.2	2.3
7	18.2	-7.04	-1	0.01	10.65	-6.43	-36.31	-10.7	-0.006	.2567	0.1	0.1	14	150	0.2	2.3
8	18.2	-7.04	-1	0.01	10.65	-6.43	-36.31	-10.7	-0.006	.2567	0.1	0.1	14	160	0.23	2
9	18.2	-7.04	-1	0.01	10.65	-6.43	-36.31	-10.7	-0.006	.2567	0.1	0.1	14	150	0.23	2

Participants in the VR paradigm validation experienced three VFDs between the movements of their actual (real) arm, and the movements of their PVA's arm within the VR environment (26.67, 200, and 400 ms). These VFDs were selected as the subset of delays used by (*13, 14*) that were associated with different patterns of lagging and leading during stable human-environment and human-human anticipatory synchronization. Regardless of the VFD presented to

participants, when $C_{D\text{-}R}$ was greater than zero the AVA movements were coupled to an individual's real time movements (i.e., with a minimum latency of 13.33 ms).

Participants experienced each D-R coupling strength × feedback delay combination twice for a total of 18 trials. The presentation of coupling strength and feedback delay conditions was organized in blocks of three trials. The presentation of delay condition combinations was randomized within two blocks of nine trials each (i.e., each coupling strength and delay condition were experienced once before any one level of each variable was repeated and each coupling strength × delay magnitude combination was experienced once before any one combination was repeated). The nine different AVA-*driver* system settings (see Table A) were also randomized within these two blocks such that each set of settings was experienced twice. Each trial lasted 60 s. The first 10 s and last 5 s of each trial was discarded to eliminate transients, resulting in 45 s trials for analysis.

Table S1.2. VR Paradigm Validation: AVA-*driver* LLE Values

Feedback Delay					
26.67 ms		200 ms		400 ms	
M	*SD*	*M*	*SD*	*M*	*SD*
.025	.013	.022	.008	.024	.016

Results

There were no significant effects of coupling strength, thus the results presented here are collapsed across this factor. Although the same nine sets of AVA-*driver* system parameters and initial conditions were used twice for all participants, the fact that AVA behavior was generated in real

time and sometimes coupled to participant movements resulted in variability in the LLEs associated with each set of parameters and conditions. Importantly, however, these values were still all positive (see Table S1.2). This confirms that the AVA-*driver* exhibited consistent chaotic movement dynamics across conditions and participants.

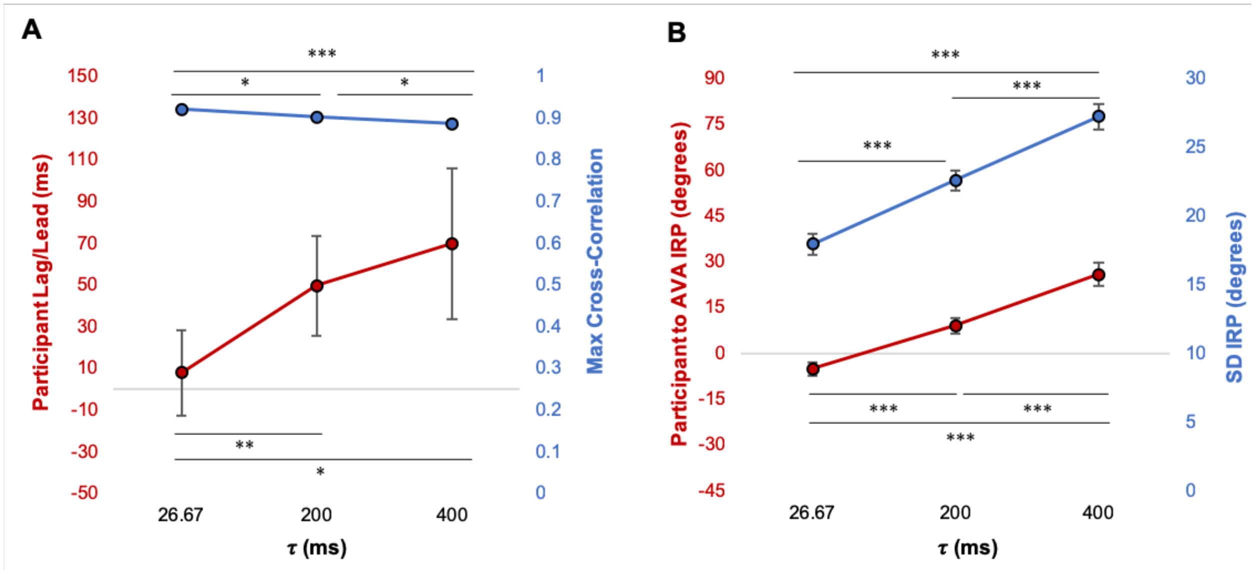

Fig S1.1. Human anticipatory synchronization during VR paradigm testing. (A) Average maximum cross-correlation (blue) and associated participant temporal lag/lead (red) (B) Mean (red) and standard deviation (blue) of instantaneous relative phase (IRP) between participant-*response* system and AVA-*driver* in each of three visuomotor feedback delay conditions. Error bars show standard error. *$p < .05$, **$p < .01$, ***$p < .001$.

As in in the primary study reported in the main text, *maximum cross-correlation* and *instantaneous relative phase* (IRP) analyses were employed to determine the occurrence of anticipatory synchronization between the response and driver systems (see the Statistical analysis section for more details). In this paradigm validation we were examining these relationships for

the AVA as *driver* and human actor as *response* system. As can be seen from an inspection of Fig S1.1, both the maximum cross-correlation and the IRP analyses revealed that increases in VFD did result in small, but non-disruptive decreases in the overall coordination observed between the participant and AVA (i.e., lower maximum cross-correlation and higher SD of IRP). More importantly, however, increases in VFD latency also resulted in a shift from near perfect synchrony to anticipation by the human actor with respect to AVA; this is captured by the average temporal lag/lead associated with the maximum cross-correlation and mean IRP, where positive lag/lead and positive IRP values correspond to the participant leading the AVA.

A one-way, within-subjects ANOVA for the maximum cross-correlation between participant and AVA movements revealed a significant effect of feedback delay, $F(1.36, 20.38) = 19.73$, $p < .001$, $\eta_p^2 = .39$ (Greenhouse-Geisser corrected). Post hoc comparisons using a Bonferroni correction revealed that all differences in average maximum cross-correlation between feedback delay conditions were significant (ps < .05), with increases in delay leading to small decreases in the coordination between the participant and the AVA-*driver*. A one-way, within-subjects ANOVA for the average temporal lag/lead associated with the maximum cross-correlation also showed a significant effect of feedback delay, here with increases in VFD leading to increased anticipation by the participant, $F(1.36, 20.38) = 9.47$, $p = .003$, $\eta_p^2 = .39$ (Greenhouse-Geisser corrected). Post hoc comparisons using a Bonferroni correction revealed significant differences in temporal lag/lead between the 26.67 ms feedback delay and both of the other VFD conditions (ps < .05).

With regards to IRP, a one-way, within-subjects ANOVA revealed a significant effect of feedback delay on mean IRP, $F(1.34, 20.13) = 177.78$, $p < .001$, $\eta_p^2 = .92$ (Greenhouse-Geisser corrected). The pattern of increased leading by the participant with respect to the AVA was very

similar to that revealed by the maximum cross-correlation analysis. Here, post hoc comparisons with a Bonferroni correction revealed that all differences in mean IRP between feedback delay conditions were significant (*p*s < .001). Finally, a one-way, within-subjects ANOVA also revealed a significant effect of feedback delay on the SD of IRP, $F(2, 30) = 76.22$, $p < .001$, $\eta_p^2 = .84$, with increases in delay leading to an increase in relative phase variability. Post hoc comparisons with a Bonferroni correction revealed significant differences in IRP standard deviation between all pairs of feedback delay conditions (*p*s < .001). As expected and consistent with (*14*) and the findings of the study reported in the main text, this increase in SD of IRP was due to intermittent phase leading and lagging behavior on the part of the participant with respect to the AVA.

Conclusions

The present findings replicated those of previous research on anticipatory synchronization in human-environment and human-human coordination (*13, 14*), thereby verifying that the current VR paradigm could be employed to investigate anticipatory synchronization. This experiment also provided a controlled context in which to evaluate the effects of bi-directional coupling on anticipatory synchronization through the use of an artificial, chaotically behaving driver system that was coupled to the coordinating behavior of human participants. This allowed us to systematically vary the influence of the human-*response* system behavior on the movements of the AVA-*driver* system in a way that has not previously been possible in the context of interpersonal anticipatory synchronization research.

The fact that the analysis measures employed here did not reveal any significant differences between any of the D-R coupling strength conditions tested (none, low, or high) is also consistent with previous work on interpersonal human anticipation (*14*). Given our ability to quantitatively

define D-R coupling in this experiment these findings actually provide strong support for the idea that bi-directional coupling (i.e., introducing D-R coupling along with the standard R-D coupling) still facilitates the emergence of anticipatory synchronization. Furthermore, the current results suggest that a human actor's awareness of the movements of a coordinating artificial agent within an HMI context does not preclude the opportunity for the artificial agent to achieve anticipatory synchronization of the human (as the results of the study reported in the main text confirm).

2. Artificial Virtual Avatar (AVA) Response System Simulations

In preparation for the real-time AVA-*response* system testing conducted in the primary study, a preliminary assessment of the AVA-*response* system was conducted through simulation. These simulations employed pre-recorded human movements as the driver system in order to determine whether it was realistic to expect the AVA to coordinate with, and potentially anticipate, naturally produced chaotic human behavior.

Two pre-recorded chaotic human movement sequences from (*14*) were used as the driver system in these simulations. In *(14)* individual participants were asked to synchronize with visually presented 2-D movement defined by fully chaotic, pre-established sequences. These sequences were generated by the same chaotic spring system used in the participant movement familiarization trials of the primary study reported in the main text. The two movement sequences chosen as driver system sequences were from two unique individuals participating in the coordination task. The LLE values for these pre-recorded aperiodic human movement (i.e., driver system) sequences are displayed in Table S1.3. They were both positive, with the data series representative of the prototypical chaotic driver dynamics produced by human participants in both the current study and in previous work. The average movement frequency for these sequences was .29 Hz (File 1: .23 Hz, File 2: .36 Hz).

Table S1.3. Pre-Recorded Human-driver LLE Values

Movement Sequence	LLE
1	0.019
2	0.058

The system of equations defining the baseline behavior of the AVA-*response* system employed was the harmonic spring oscillator as described in Eq. 2 of the main text. Six different R-D coupling values, $C_{R\text{-}D}$, (1, 1.5, 1.75, 2, 2.25, and 2.5) and seven delay latencies, τ, (<16.66, 16.66, 33.32, 99.96, 199.92, 299.88, and 399.84 ms) were tested.

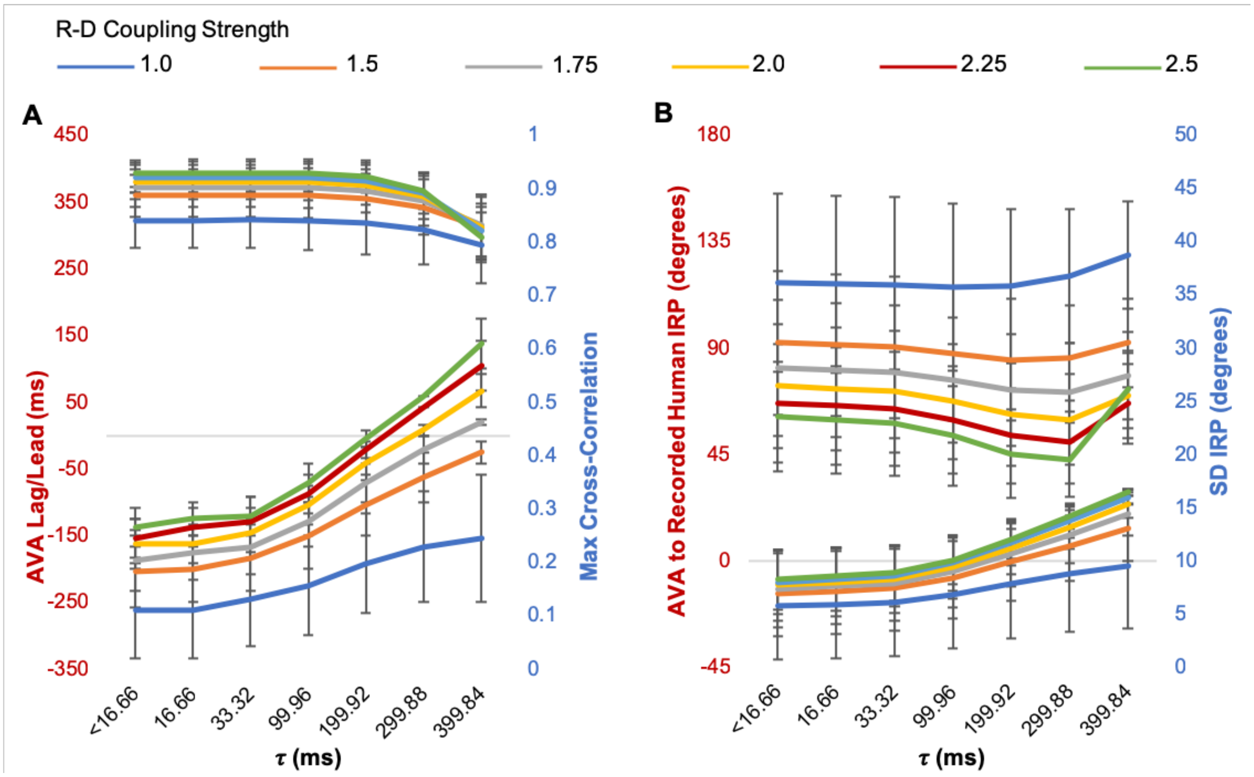

Fig S1.2. AVA-response anticipatory synchronization during simulation with pre-recorded aperiodic human-driver movement. (A) Average maximum cross-correlation (blue) and associated AVA temporal lag/lead (red) and (B) Average IRP (red) and standard deviation of IRP (blue) between AVA and human movement series for each combination of the six visuomotor self-referential AVA delays and seven R-D coupling strengths. Error bars show standard error.

The coupling strength term, C_{R-D}, was set to the value for a given simulation prior to the beginning of the simulation. The influence of delayed feedback coupling within the AVA was introduced after the amount of time equal to the delay latency for that simulation had passed (i.e., enough response system behavior had to be generated in order for the current state of the system to reference its' past behavior). Each coupling strength × delay condition was simulated once for a total of 42 simulations per driver system sequence. For the sake of consistency, each simulation was 60 s long and was conducted at 60 Hz using the same Unity 3D C# code environment employed for the primary study.

Results

The same maximum cross-correlation and IRP analyses employed above and in the primary study reported in the main text were employed to index the degree to anticipatory synchronization observed in the AVA-*response* system simulations. As can be seen from an inspection of Fig S1.2, these simulations provided corroborating evidence that the AVA-*response* system developed for the current study was capable of achieving anticipatory synchronization with respect to chaotic human behavior. More specifically, the temporal lag/lead associated with the maximum cross-correlation analysis revealed that anticipatory behavior was observed for feedback delays above 299.88 ms, particularly for the three highest R-D coupling strengths (C_{R-D} = 2.0, 2.25 and 2.5). The IRP analysis further revealed similar anticipatory synchronization results. The mean IRP results revealed AVA leading behavior at the longest two AVA self-referential feedback delays (299.88 and 399.84 ms) for all R-D coupling strength conditions tested above C_{R-D} = 1.0, and at the 199.92 ms feedback delay for the four highest R-D coupling strengths (C_{R-D} = 1.75, 2.0, 2.25

and 2.5). Small magnitudes of anticipatory synchronization were even observed at the 99.96 ms feedback delay for the highest R-D coupling strength (C_{R-D} = 2.5).

Conclusions

It is important to note that although the magnitude of anticipatory behavior observed for these simulations is less pronounced than that exhibited in previous 'model-only' simulations (*8, 20)*, this is likely due to the presence of non-deterministic (i.e. stochastic) movement variability within the human-movement time series employed. Indeed, the patterns of leading, lagging, and intermittent coordination exhibited by the AVA-*response* system with respect to the pre-recorded chaotic human behaviors was overall similar to those previously observed in studies of anticipatory synchronization during human-environment and human-human coordination (*13, 14*). Ultimately the HMI anticipatory synchronization findings presented in the main text were also consistent with the findings of the simulations and paradigm validation results presented here, as well as previous work on feedback-delay induced anticipatory synchronization.
